# Is countries’ transparency associated with gaps between countries’ self and external evaluations for IHR core capacity?

**DOI:** 10.1186/s12992-020-0541-3

**Published:** 2020-01-20

**Authors:** Feng-Jen Tsai, Battsetseg Turbat

**Affiliations:** 1 PhD Program in Global Health and Health Security, College of Public Health, Taipei Medical University, 250 Wu-Hsing Street, Taipei City, 110 Taiwan; 2 Master Program in Global Health and Development, College of Public Health, Taipei Medical University, Taipei, Taiwan

**Keywords:** Transparency, International health regulations 2005 (IHR 2005), Global health security, Self-evaluation, Joint external evaluation (JEE), Civil liberties, Human development index (HDI)

## Abstract

**Background:**

This study aims to evaluate the gap between countries’ self-evaluation and external evaluation regarding core capacity of infectious disease control required by International Health Regulations and the influence factors of the gap.

**Methods:**

We collected countries’ self-evaluated scores (International Health Regulations Monitoring tool, IHRMT) of 2016 and 2017, and external evaluation scores (Joint External Evaluation, JEE) from WHO website on 4rd and 27rd November, 2018. There were 127 and 163 countries with IHRMT scores in 2016 and in 2017, and 74 countries with JEE scores included in the analysis. The gap between countries’ self-evaluation and external evaluation was represented by the difference between condensed IHR scores and JEE. Civil liberties (CL) scores were collected as indicators of the transparency of each country. The Human Development Index (HDI) and data indicating the density of physicians and nurses (HWD) were collected to reflect countries’ development and health workforce statuses. Then, chi-square test and logistic regression were performed to determine the correlation between the gap of IHRMT and JEE, and civil liberties, human development, and health workforce status.

**Results:**

Countries’ self-evaluation scores significantly decreased from 2016 to 2017. Countries’ external evaluation scores are consistently 1 to 1.5 lower than self-evaluation scores. There were significantly more countries with high HDI status, high CL status and high HWD status in groups with bigger gap between IHRMT and JEE. And countries with higher HDI status presented a higher risk of having bigger gap between countries’ self and external scores (OR = 3.181).

**Conclusion:**

Our study result indicated that countries’ transparency represented by CL status do play a role in the gap between IHR and JEE scores. But HDI status is the key factor which significantly associated with the gap. The main reason for the gap in the current world is the different interpretation of evaluation of high HDI countries, though low CL countries tended to over-scored their capacity.

## Introduction

Infectious disease is one of the most significant health and security challenges for the world damaging global economics and public health [1–3]. After the SARS pandemic in 2003, International Health Regulations 2005 (IHR 2005) were adopted by the World Health Organization (WHO) to enhance the global capacity to prevent and control infectious diseases [4]. One of the approaches adopted by IHR 2005 is to require member states to develop minimal core public health capacities to implement the IHR 2005 effectively.

To monitor progress in this regard, WHO introduced a self-assessment process for countries to report on their implementation of IHR 2005 [5]. The IHR Secretariat at WHO developed the IHR Core Capacity Monitoring Framework and released the IHR Monitoring Tool (IHRMT) to monitor progress in implementing IHR core capacities in 2010 [6]. With this standardized data collection tool, countries were recommended to fill out the IHRMT and submit completed reports to WHO annually [7].

This self-report process received such insufficient attention that in 2014, only 60 countries reported their self-assessment to WHO. The responses of the 2014 to 2016 Ebola outbreak in West Africa have resulted in a multitude of review panels, many of which agreed that the self-assessment process was flawed - in that it did not necessarily reflect an accurate picture of national capacity for disease control [8–10]. With this weakness, the review panels recommended a shift of mechanisms from the self-report to Joint External Evaluations (JEE) concerning national capacities in pandemic preparedness [11].

Previous study has found that though IHR self-evaluation and JEE are evaluated the same capacity for infectious disease control, JEE scores were found out to be approximately one step lower than countries’ self-reported IHRMT [12]. But up to now, there is no study focused on the reason for the gap between IHR self-evaluation and JEE scores.

If the main reason for the gap is the different understandings of Indicators, the problem could be solved by concentrating the discussion of conceptualization and operationalization of the tools. However, if the cause of the gap mainly came from the objective attitude of the countries like over-reporting scores for more funding, then we might have to rethink about the need and the effectiveness of this evaluating approach and if it is appropriate to allocate resource based on the outcome [13, 14].

For clarifying if the objective attitude of the countries is the main reason for the gap, we conducted this study with the hypothesis that countries with better transparency would have less risk of having big gap between countries’ self-evaluation and external evaluation.

The most pressing rationale for transparency in evaluation of national infectious disease capacity is that open communication and information can enhance the public supervision for preventing the manipulation of the assessment outcome [15–19]. The concept of transparency can be represented by the extent of civil liberties and the circulation of public information [16]. Civil liberties are the basic tenets of democracy that indicating the rights and freedoms which protect individuals from unfair infringement by the government of the nation where they reside [16]. Civil liberties further set limits on the government from abusing their power and interfering unduly with the affairs of private citizens. Countries with strong civil liberties typically also have well-developed mass media that is capable of reporting news regarding infectious disease control. With personal safety and security, there would be better space for public supervision for government movement [20]. Therefore, the countries’ self-evaluation and external evaluation would be more accurate based on accountable information, and the gap between countries’ self-evaluation and JEE would be smaller.

With above assumption, we conduct this study in order to understand the association between countries’ transparency and the gap between self and external evaluation scores for IHR core capacity.

## Methods

We applied the methodology developed and used in previous study which indicating the important role of transparency in the gap of reporting timeliness in infectious disease [21]. While Systemic Rapid Assessment (SYSRA) is a framework includes External contexts as social-environmental factors and health-specific elements which echoing the element of national responsibilities required by IHR 2005, it was consulted to be the conceptual and analytical guidelines for the evaluation of health systems and infectious disease control programs [22, 23]. Therefore, we collected transparency data and measurements based on this framework for further analysis.

### IHRMT (self-evaluation) and JEE (external evaluation)

IHRMT is a questionnaire to monitor progress in implementing the IHR of countries [5]. The questionnaire consists of 13 sections including 8 core capacities, points of entry and 4 ‘other hazards’ as identified and delineated by the WHO to match the obligations outlined in Annex 1 of the IHR. Eight core capacities mainly for infectious disease control include legislation, coordination, surveillance, response, preparedness, risk communication, human resources and laboratory. The 4 hazards include zoonosis, food safety, along with the chemical and radionuclear ones. Individual questions were grouped by components and indicators in the questionnaires including 256 total attributes.

The JEE is a data gathering instrument designed to evaluate a country’s capacities for health security, including all IHR core capacities across relevant sectors at a national level [24]. The tool has 19 technical areas that includes the core capacities identified by IHRMT. The JEE also includes capacities specially identified for health security, such as Antimicrobial Resistance, Biosafety and Biosecurity, Immunization, Emergency Response Operations, Linking Public Health and Security Authorities and Medical Countermeasures and Personnel Deployment. The JEE process involves a self-evaluation by the country, followed by an external assessment team visit, that then produces a full JEE report that includes scores for the Indicators, as well as identified priority actions.

### Data collection

We obtained countries’ self-reported implementation percentages as scores from the WHO website on 4rd November 2018 [25]. There were 127 countries’ self-reported IHRMT (now advanced to be IHR Self-Assessment Annual Reporting Tool, SPAR) scores in 2016 and 163 countries’ self-reported IHRMT scores in 2017 available and used in the study. Seventy-four countries’ published JEE reports were further collected on 27th November, 2018 and used for analysis [26].

The average score of 8 core capacities was further calculated to represent overall national capacity regarding infectious disease control.

### Measurements

Civil liberties scores from the Freedom House were collected as indicators of transparency for each country. The Freedom House is an independent nongovernmental organisation that dedicating to the expansion of democracy and freedom around the world [27]. This group annually evaluates the political rights and civil liberties of each country. In our study, we used only civil liberties as an index of transparency. Civil liberties which reviewed by a 15 questions checklist included 4 key areas: freedom of expression and belief (4 questions), associational and organisational rights (3 questions), rule of law (4 questions) and personal autonomy and individual rights (4 questions). The total number of points on the civil liberties checklists will further be transformed into a rating scale ranged from 1 to 7. Score 1 represents the highest degree of freedom and 7 represents the lowest degree. The details of the method are described in the methodology section of the Freedom House website [27]. We collected the civil liberties scores of 2016 and further divided the analysed countries into free, partly free and not free countries according to these scores. Countries with civil liberties scores of 1 and 2 were designated as free countries, countries with scores of 3 to 5 were considered partly free countries, and countries with scores as 6 and 7 were not free countries.

Based on the framework of SYSRA toolkit, we further searched the Human Development Index (HDI) from the United Nations Development Program (UNDP) and information regarding the density of physician and nurses from WHO o represent the general health capacity of the country [28, 29].

According to the definition, Human development encompassed three dimensions: life expectancy at birth which indicating population health and longevity; adult literacy rate which indicating the knowledge and education level and the gross domestic product per capita indicating the purchasing power parity. With indicators mainly collected from official statistics, the human development index was calculated as a simple average of the dimension indices ranging between 0 and 1, with 1 representing the highest degree of human development and 0 the lowest. The details of methods are described in the Technical Notes section of the report [30]. We used the human development index of 2016 to represent the human development status of each country in that year. In addition, the categories used by the UN, i.e., very high, high, medium and low development countries were also used in the study.

Information of each country’s density of physicians and nurses was collected from WHO websites [31]. Then the sum of these two scores was calculated and used as the index of the health workforce in the study. We then categorized countries into high, middle or low health workforce countries based on the sum of the density of physicians and nurses in each country. Countries with upper tertile scores of health workforce density were defined as countries with high health workforce. Countries with the middle and lower tertile scores of health workforce density were defined as middle and low health workforce countries, respectively.

### Analysis

The response for IHR from countries comprises the percentage of implementation ranging from 0 to 100. The JEE is scored on a scale from 1 to 5 to represent the level of a countries’ capacity to meet an indicator of health security, with 5 being the highest level of capacity. In order to make the IHR and JEE scores comparable, we re-scored the IHR results by dividing the scores by 20 to condense the scores into the scale of 5. We then calculated the difference of average score of each item between IHR self-evaluation and JEE to represent the gap between the different evaluation approaches. And the gap is further divided into 2 groups (less gap v.s. big gap) with average as cutting point. Similarly, the score gap between IHR 2016 and IHR 2017 is divided into 3 groups (negative gap, no gap, positive gap) for further analysis. The negative gap groups refers to countries with lower IHR 2017 scores than IHR 2016.

Pair-t test is used to compare the score of each item between IHR 2016 and IHR 2017, IHR 2016 and JEE, and IHR 2017 and JEE as it represent countries’ original self-judgment of their capacity without external interference. Chi-square test was then applied to compare countries’ HDI, civil liberty, health workforce between the gap group of IHR 2016 and IHR 2017, IHR 2016 and JEE, IHR 2017 and JEE. And we further compare countries’ HDI, civil liberty and health workforce between the gap group of countries’ first IHR self-evaluated score and JEE scores. Logistic regression was then applied to evaluate the association between countries’ HDI, CL and the gap between countries’ first IHR and JEE. Though HDI, CL and HWD were all significantly different between the gap groups of countries’ first IHR and JEE, we included only HDI and Cl in the regression analysis due to the fact that HDI and HWD was significantly correlated by Person correlation coefficient test.

All analysis was performed using the software SPSS, Version 18.0.

## Results

### IHR self-reported scores in 2016 and 2017, and JEE external-evaluated scores

**Fig. 1:**
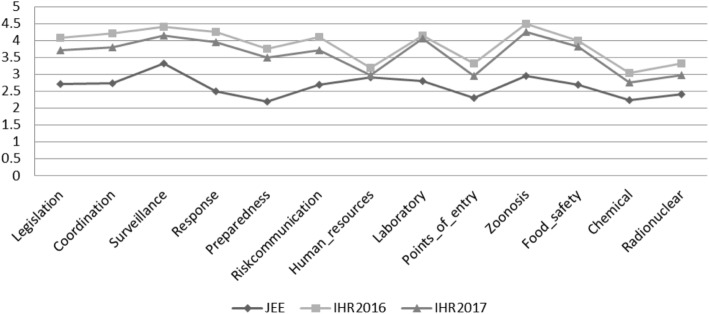
IHR self-reported scores in 2016 and 2017, and JEE external-evaluated scores

Scores of IHR core capacities reported by country in 2016 and 2017 and scores of JEE are shown in Table 1. And the compressed score of IHR 2016 and IHR 2017 and the score of JEE is illustrated in Fig. 1.

**Table 1:** the core capacities of IHR2017, 2016 and JEE

Scores	IHR 2016			IHR 2017			JEE		
	*N* = 127			*N* = 163			*N* =74		
	Mean	Range	SD	Mean	Range	SD	Mean	Range	SD
Legislation	81.5	0-100	29.25	73.47	0-100	33.87	2.72	1-5	1.24
Coordination	84.14	20-100	20.62	76.13	0-100	28.44	2.74	1-5	1.23
Surveillance	87.72	30-100	13.87	83.01	25-100	17.12	3.31	1.5-5	0.66
Response	85.07	0-100	17.75	79.06	6-100	21.81	2.5	1-4.83	1.13
Preparedness	75	0-100	25.91	69.55	0-100	28.7	2.2	1-5	1.3
Riskcommunication	81.31	14-100	23.11	73.72	0-100	28.83	2.7	1-4.4	0.81
Human_resources	63.36	0-100	32.8	59.63	0-100	32.4	2.9	1-5	0.94
Laboratory	82.98	25-100	18.55	81.2	17-100	20.13	2.8	0-4.83	0.9
Points_of_entry	66.33	0-100	32.2	59.04	0-100	34.8	2.3	0-5	1.3
Zoonosis	89.37	22-100	17.9	84.94	0-100	22.1	2.94	1-5	0.97
Food_safety	80.09	0-100	26.11	76.41	0-100	27.02	2.7	0-5	1.33
Chemical	60.53	0-100	34.6	55.28	0-100	34.53	2.23	1-5	1.23
Radionuclear	66.45	0-100	34.73	59.46	0-100	36.54	2.4	1-5	1.25

Among the 127 countries which had IHR scores in 2016, the average score of all the indicators ranged from 60.53 to 89.37. Among 163 countries had IHR scores in 2017, the average score of all indicators ranged from 55.28 to 84.94. Pair-t test result showed that scores of all items in 2017 are significantly lower than scores in IHR 2016.

The trend of average scores of IHR and JEE were parallel, except human resource. And IHR 2016 scores were the highest while JEE scores were lowest among all indicators. Pair-t test showed that the differences of all the items between IHR 2016 and JEE were statistically significant, except Human resources. And the differences of all the items between IHR 2017 and JEE were also statistically significant, except Human resources and Points of Entry.

### Comparison of HDI, civil liberties and health workforce between IHR different gap groups by chi-square

The comparison of HDI, CL and HWD between 3 gap groups between IHR 2016 and IHR 2017 are showed in Table 2. From the analysis, HDI and health workforce (HWD) were significantly different between groups. The difference of civil liberties was also close to statistically significant between groups (*p* = 0.056).

**Table 2:**
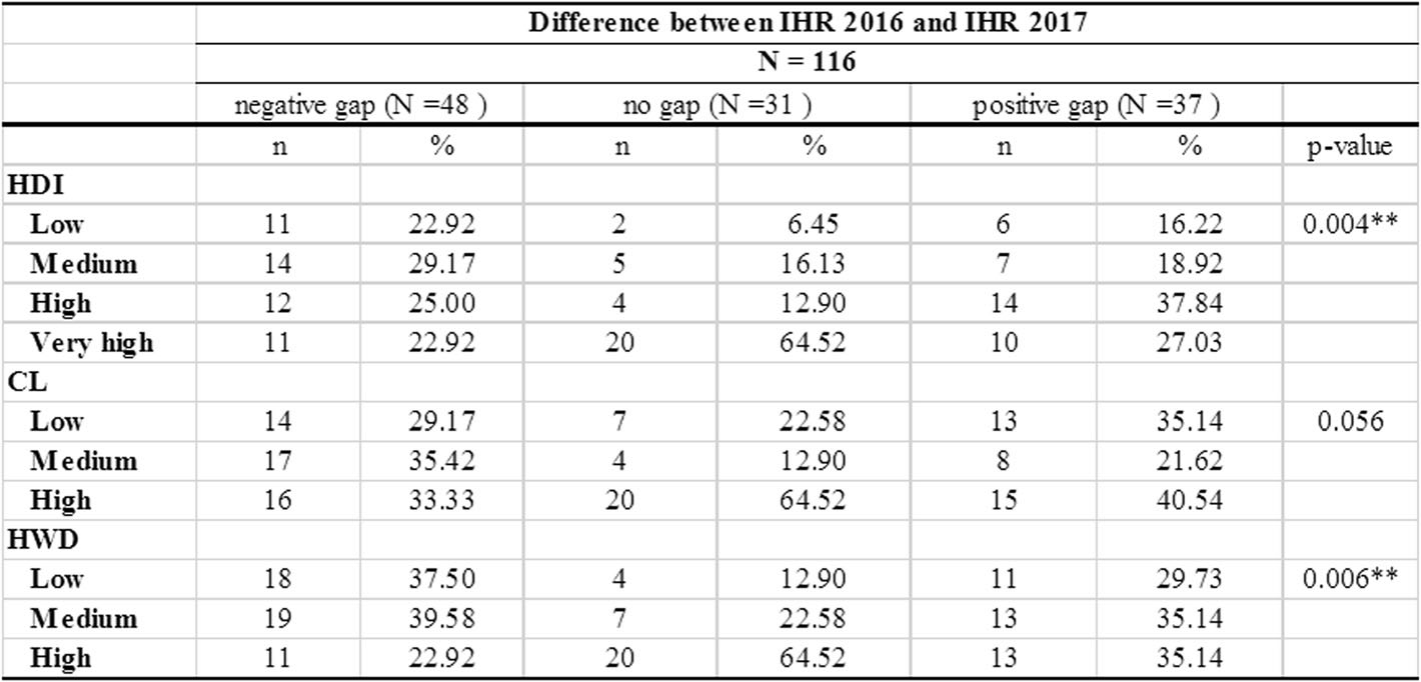
Comparison of HDI, CL and HWD between different IHR gap groups

Overall speaking, there were 52% low and middle HDI countries in negative gap group while the percentage of high and very high HDI status countries were 65% in positive gap group. And there were 77% countries with low and middle HWD status in negative gap group while around 35% of high HWD status countries were in positive gap group. For civil liberty, there were approximately 65% of countries with low and middle CL status in negative gap group while there were 40% high CL status countries in positive gap group.

### Comparison of HDI, civil liberties and health workforce between the gap groups between IHR and JEE by chi-square

Comparison of countries’ HDI, CL and HWD between different gap groups between IHR and JEE are showed in Table 3. General speaking, there were more countries with low and middle HDI status, low and middle CL status and low and middle HWD status in less gap group between IHR and JEE, no matter between IHR 2016 and JEE, between IHR 2017 and JEE, and countries’ first IHR and JEE. In contrast, countries with high HDI status, high CL status and high HWD status countries were more in big gap group between IHR and JEE.

**Table 3:**
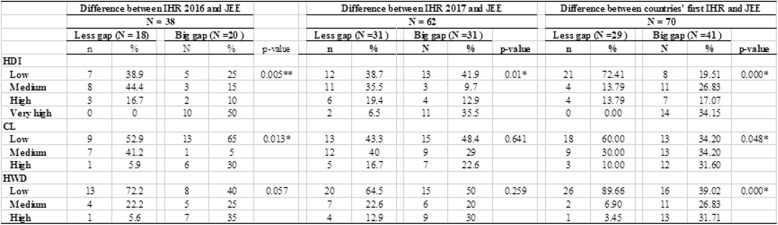
Comparison of HDI, CL and HWD between different gap groups between IHR and JEE

Between IHR 2016 and JEE, HDI and CL were significantly different between less and big gap groups. And health workforce was close to being significantly different (*P* = 0.057) between groups. There were 83% low HDI countries in less gap group while there were 60% high HDI countries in big gap group. For civil liberty, there were only 5.9% high CL countries in less gap group while there were 30% high CL countries in big gap group.

Between IHR 2017 and JEE, HDI was statistically different between less gap and big gap groups. The percentage of countries with low and medium HDI (74.2%) status was high among less gap group. In contrast, the percentage of countries with very high HDI (35.5%) status was high among big gap group.

Between countries’ first IHR and JEE, HDI, health workforce and civil liberties were all statistically different between less gap and big gap groups. The percentage of countries with low HDI (72%), low CL (60%) and low HWD (89%) were all higher in less gap group than big gap group. In contrast, the percentage of countries with very high HDI (34%), high CL (31%) and high HWD (31%) status was high among big gap group.

### Association between HDI, civil liberties and the difference between countries’ IHR and JEE scores by logistic regression

Table 4 showed the correlation of HDI, civil liberties and the difference between countries’ first IHR and JEE. Result of logistic regression indicated that HDI, was significantly associated with the gap between countries’ first IHR and JEE scores. Countries with higher HDI status presented a higher risk of having bigger gap between countries’ IHR and JEE (OR = 3.181 (95% CI: 1.71, 5.93)).

**Table 4:** Associations between HDI, CL and gaps between countries’ first IHR and JEE

	Difference between IHR 2016 and JEE	
	*β*	ORs (95% CI)
HDI	1.157	3.181 (1.71-5.93)***
CL	-0.368	0.69 (0.30-1.57)

## Discussion

This is the first study which focused on the reason behind the gap between countries’ self and external evaluation in national infectious disease control capacity required by IHR 2005. The difference between JEE and IHR 2016 and IHR 2017 were both significant though IHR 2017 is already significantly lower than IHR 2016. Further analysis showed that HDI, HWD is significantly different between the gap groups between IHR 2016 and JEE. And HDI, CL and HWD were all significantly different between the gap groups between countries’ first IHR and JEE. Regression analysis showed that countries with higher HDI status had 3 times higher risk of having bigger gap between countries’ IHR and JEE.

From our study, most of the countries downgraded their self—evaluated IHR scores from 2016 to 2017. And the difference between IHR 2016 and IHR 2017 is significant. This phenomenon might reflect the impact of JEE that countries adjusted theirs self-evaluated scores based on the consensus regarding the evaluation standard due to the conduct of JEE. There is also a possibility that countries downgraded their IHR scores due to the pressure of external evaluation. While the external experts will review their capacity on-site, countries might consider reducing the over-report of appropriate behaviors. The changing nature of financing health security, especially insufficient financing in preparedness, might also be the reason for the changes of the score. The JEE and self-reporting scores are fluid in nature as lack of financing can result in downgrading of scores as well. Further qualitative study is recommended to understand the reason behind.

From study finding, the Human Resources was the only item without significantly different gap between self and external evaluations for both years. One of the explanations for this phenomenon is the nature of the indicator and the evaluating way of the tool. All the countries had health professionals and field epidemiology training program, no matter the quantity and the quality is sufficient enough or not. In addition, human resources is one of the fundamental and prioritized item for each country when they developing or strengthening the health system. Human resource training is also the prioritized area for foreign health aid. Therefore, the gap in human resources is much smaller than others.

While most low and middle HDI and low HWD countries downgraded their scores in IHR 2017, countries with high HDI, HWD and high CL upgraded their scores which indicating the improvement of their capacity. This phenomenon might also reflect the need of assistance for low HDI and low HWD countries around the world. Though those countries recognized the need for strengthening their core capacity and more clear about the gap from the process, they are lack of sufficient resources to improve the capacity. Further discussion regarding the issue is needed.

From the study, the gap between IHR and JEE was bigger among countries with high HDI. One of the possible explanations for this phenomenon is that countries with high HDI status have more resources and capacity for related research and development. And so, there are many experts in each capacity field required by the tool. While experts might hold different viewpoints and standards regarding the evaluation approach in the early development stage of the tool, the gap between internal and external evaluation is bigger. While most low HDI countries had insufficient resources for establishing the core capacity for infectious disease control, the score of self-evaluation and JEE would be similarly low. And so, the gap between the JEE and self-reported score is smaller for low HDI countries. Further followed study is needed to see if the gap between high HDI countries is reduced for consensus of the tool.

From the study, we also found that CL status is relevant to the gap between IHR 2016 and IHR 2017, and countries’ first IHR and JEE. The result showed U-shape relationship between countries’ CL status and their gap between IHR and JEE. Countries with high CL status and countries with low CL both had higher chance to have big gap between their IHR score and JEE score. While the countries with high CL status, high HDI and HWD status were usually the developed countries with leading position in global health, the different viewpoint of the evaluation standard might be the explanation for the phenomenon. And this gap might be reduced with the development of JEE 2.0. Differently, there were still countries with low CL status continuously scored themselves as 100 for more than 80% of the items though their HDI and HWD status were consistently low in years.

There are several limitations of the study. First, our study was cross-sectional in nature, so the findings of our study can only be considered to be an association rather than a causal relationship. Second, Moreover, the results of our study might overemphasise the effect of transparency on the gap between countries’ self and external evaluation because we were not able to analyse other factors related to the gap, such as communication infrastructure and materials provided for evaluation.

## Conclusion

In conclusion, our study result indicated that countries’ transparency represented by CL status do play a role in the gap between IHR and HEE scores. But HDI status is the key factor which significantly associated with the gap. In other words, the main reason for the gap between IHR and JEE in the current world is the different interpretation of core capacity evaluation of high HDI countries though low CL countries tended to over-scored their capacity. The situation could be improved while JEE provided opportunity for experts to increase the related discussion. Further studies are needed to understand the impact and outcome of JEE 2.0.

## Data Availability

All the data in this research are obtained from publicly available sources.

## Abbreviations

CL: Civil liberties
HDI: Human development index
HWD: Health workforce density
IHR: International health regulation
IHRMT: International Health Regulations Monitoring tool
JEE: Joint external evaluation
UN: United Nation
UNDP: United Nations Development Program
WHO: World Health Organization
